# Trauma and identity predictors of ICD-11 PTSD and complex PTSD in a trauma-exposed Colombian sample

**DOI:** 10.1177/00207640251318074

**Published:** 2025-02-28

**Authors:** Martin Robinson, Emanuele Fino, Gülseli Baysu, Rhiannon N Turner, Natasha I Bloch, Donncha Hanna, Chérie Armour

**Affiliations:** 1 School of Psychology, Queen’s University Belfast, Northern Ireland, UK; 2 Department of Biomedical Engineering, Universidad de Los Andes, Bogota, Colombia

**Keywords:** Psychological trauma, mental health, identity, risk, resilience

## Abstract

**Background:**

The 11th International Classification of Diseases introduces the diagnosis of Complex PTSD (CPTSD); characterized by traditional PTSD symptomology plus Disturbances in Self Organisation. Part of this construct involves feeling socially disconnected from others, suggesting that aspects of group and individual identity may be associated with this disorder.

**Aims:**

The current study seeks to contribute to better understanding the association of individual social and personal identity in development of this disorder in post-conflict contexts.

**Methodology:**

This study analysed survey data collected as part a case-control investigation of psychological risk and resilience in a trauma-exposed sample in Colombia (*N* = 541). Identity orientations, that is, the level of importance ascribed to one’s social and personal identity, was assessed using the Social and Personal Identities Scale (SIPI) and was assessed as predictor of probable CPTSD diagnosis using multinomial logistic regression.

**Results:**

Analyses indicated that trauma experiences were associated with both diagnostic categories, however Social and Personal identity orientation were significant predictors of probable CPTSD diagnosis, but not probable PTSD diagnosis. Greater Personal identity orientation, that is, viewing oneself as individualistic, was associated with increased likelihood of CPTSD. In contrast, greater Social Identity orientation, that is, stronger group membership identification, was associated with reduced odds of CPTSD diagnosis. Identifying as a victim of the conflict was not significantly associated with risk for PTSD or CPTSD outcomes.

**Conclusion:**

Greater sense of Social Identity and cohesion is suggested to be protective against CPTSD development, whereas greater personal identity orientation is a risk factor. Theoretical perspectives considering the role of social and personal identity may be valuable in understanding individual risk for CPTSD in post-conflict societies.

## Introduction

A prolonged period of civil conflict has taken place in Colombia resulting in a widespread experience of Potentially Traumatic Events (PTE’s) (Internal Displacement Monitoring Centre [IDMC], 2023; Shultz et al., 2014). Those living in post-accord contexts are at increased risk for traumatic stress outcomes (Eide & Dyrstad, 2019). Attributable to a history of conflict trauma and displacement associated with socio-political identities that divide various factions, Colombia is argued to possess a people with varied identities that pose a challenge to integration and mental health (Cuartas & Roy, 2019; Gluecker et al., 2021).

There are a myriad of factors that may promote risk and resilience among those who experience PTE’s across the biological, psychological and social spectrum (Iacoviello & Charney, 2014). While all aspects of the biopsychosocial framework may be useful in understanding the response to traumatic stress, it is suggested that those psychosocial factors that confer additive risk for mental ill-health following trauma may be considered of particular importance as they potentially represent more viable intervention targets. Indeed, there has been a call to examine the role of the social environment both as a consequence of traumatic stress, and as potential facilitator of resilience and recovery (Haslam et al., 2008; Muldoon et al., 2017).

One of the critical factors in the social environment is the extent to which one identities with their community. It has been suggested that when acute adversity is experienced individuals may experience greater social cohesion and group support that ultimately reduces the likelihood of distress outcomes and promotes resilience (Mancini, 2019). In this instance, individuals may paradoxically experience greater support through collective stressors, and those with more collective or social group orientation may be at a decreased risk for traumatic stress outcomes. Collective identity and orientation may therefore act as a salient protective factor for traumatic stress outcomes.

Existing evidence has robustly supported perceived importance of social identity, support, and embeddedness as protective factors following PTE’s (Wagner et al., 2016). Specifically, those who perceive themselves to have greater social identification and support perceive greater personal resources (e.g. esteem, cohesive sense of self), which in turn alleviates the stress experienced (Hobfoll et al., 1990). Indeed, in a meta-analysis of 67 studies, Wang et al. (2021) evidenced an enduring stress-buffering effect of perceived social support following trauma exposure, meaning that this may act as a robust and persistent resilience promotion factor. Greater orientation to social or group identification may confer similar benefits, indicative of resources of support one may draw on to alleviate psychological distress (see Jetten et al., 2012).

An additional consideration is the effect of traumatic stress on the formation and dissolution of group identity. Muldoon et al. (2020) outlined the *Social Identity Model of Identity Change*, proposing that following trauma individuals may experience changes in aspects of their sense of self and that these changes may contribute to psychopathology. In this model, traumatic stress may disrupt an individual’s sense of identity and group connection, which undermines mental well-being. This aspect of the ‘social curse’ hypothesis also holds that identification with groups that are associated with adversity may act as a risk factor for traumatic stress if group membership is stigmatised (Jetten et al., 2012; Lashkay et al., 2023). Indeed, those with victim status (self-identified as a victim of armed conflict) in Colombia are considered to be at risk for adverse mental health outcomes due to experience of potentially traumatic events characteristic of this group (Tamayo-Agudelo & Bell, 2019). The effects of group identification associated with potentially traumatic events must therefore be considered.

Personal identity orientation, in contrast, is envisioned as an identity domain that differentiates the individual as distinct from others, for example, feeling unique and original when compared to others (Nario-Redmond et al., 2004). In this domain individuals place greater importance on individualistic maintenance of self-concept and wellbeing, for instance, it is theorised that those more individualistic in identity draw more satisfaction and support from personal goal achievement and rely less on the support of others to promote personal wellbeing (Oyserman et al., 2002).

Much extant literature has focused on Posttraumatic Stress Disorder (PTSD) as the principal pathological outcome following traumatic stress, however the most recent iteration of the International Classification of Diseases includes a new diagnostic category of *Complex Post-Traumatic stress Disorder* (CPTSD), defined by PTSD symptoms with additional *disturbances in self-organisation* (WHO, 2024b). These additional symptoms specifically include disturbed interpersonal relationships characterised by ‘*persistent difficulties in sustaining relationships and in feeling close to others*’ (WHO, 2024a, Section 6B41 para. 1) which may be considered on its face to relate to identity and social group embeddedness. Indeed, previous research has suggested that those who experience interpersonal trauma may struggle to integrate a coherent sense of identity (Kouvelis & Kangas, 2021). The role of internal and group identity may therefore be considered of particular importance in the development and expression of this disorder as individuals may draw on social support resources mitigating *disturbances in self-organisation* and *relationships* characteristic of CPTSD (see Hyland et al., 2023).

Alternatively, personal identity orientation may be protective as avoidance behaviours characteristic of PTSD are more consistent with personal or individualistic identity orientation, and thus potentiallty less disruptive to those individuals (Jayawickreme et al., 2013). Greater ascribed personal identity orientation may also act as a risk factor for CPTSD outcomes as this may suggest foregoing the support garnered from a shared identity that has been demonstrated to be protective (Muldoon et al., 2020), and more directly exacerbate *disturbances in interpersonal relationships* symptomology. The experience of psychological trauma, and integration thereof into one’s personal self-concept, is argued to be a potential risk/resiliency factor associated with PTSD (Kouvelis & Kangas, 2021).

Likewise, of great importance is understanding risk and resilience factors for CPTSD diagnosis. Previous evidence has highlighted interpersonal trauma, and greater cumulative exposure to trauma events as salient risk factors for this condition (Cloitre et al., 2009; Hyland et al., 2017; Knefel et al., 2019). It has however been noted that extant evidence for risk factors associated with CPTSD is primarily drawn from US and UK samples, with fewer examining these in non-western, industrialised, global north, samples (Kvedaraite et al., 2022). Accordingly, we will examine whether trauma burden, that is, exposure to multiple traumatic events, may predict the likelihood of reporting CPTSD, hypothesising that experience of interpersonal and greater cumulative trauma events will be associated with CPTSD.

Emergent review and meta-analytic evidence of risk factors has indicated that being female and having limited economic resource (e.g. through unemployment, low socioeconomic status) are positively associated with CPTSD (Leiva-Bianchi et al., 2023). It has been suggested that these groups are also more likely to experience potentially traumatic events considered risk factors for CPTSD (Ho et al., 2021). However, across studies it has been shown that these groups, for example, females, are not more likely to report trauma event exposure but are more likely to report complex traumatic stress (Leiva-Bianchi et al., 2023). It is therefore suggested that sociodemographic factors, and experiences associated with them, may be associated with CPTSD risk. This study thus seeks to evaluate the association of trauma exposure and demography associated with CPTSD in this Latin American sample.

It is argued that social identity in relation to the nascent concept of CPTSD, containing specific symptoms of disturbed interpersonal relationships, may be of particular importance. It is hypothesized that those who report greater levels of social identification will be less likely to report CPTSD pathology as they engage more with social supportive resources. Conversely, it is hypothesized that those who place greater importance on their individualistic identity will be more likely to report CPTSD pathology as difficulties such as disturbed relationships are exacerbated by the interaction of pathology and this identity orientation.

Extant evidence thus suggests that identity orientation may act as a social cure or curse associated with PTSD (Jetten et al., 2012). Evidence is however critically lacking regarding identity as potential risk and resiliency factor for CPTSD, and study warranted given the potential relevance for expressions of disturbances in self-organisation characteristic of this disorders. The current study thus aimed to; (1) explore the association between trauma events and cumulative lifetime trauma with probable PTSD and CPTSD diagnosis, and (2) examine identity predictors (social and personal identity orientation, victim identity) of PTSD and CPTSD in this trauma-exposed Colombian sample.

## Methodology

### Data and Sample

These data were drawn from the MI-VIDA case-control study. This study investigates biopsychosocial risk factors associated with PTSD in a sample of trauma-exposed community residents living in the northern region of Colombia; Cesar and Atlantico Departments. The wider investigation aimed to survey this cohort of individuals at three time points over 12 months assessing a myriad of risk and resiliency factors associated with traumatic stress outcomes. The current investigation analyses the baseline dataset collected between February 2022 and July 2023.

The baseline dataset contained *N* = 562 total records. Among these there was 2.1% item-level missingness across *n* = 59 records, found to be Missing Completely at Random [Little’s MCAR test: χ² = 1606, df = 1961, *p* > .999]. Missing data was estimated for those cases with item-level missingness of 20% or less, a principled cut off to estimate missing value when data are MCAR, using the Expectation Maximisation algorithm (Dong & Peng, 2013). This resulted in a final effective study sample of *n* = 541 participants.

### Measures

*Sociodemographic* were measured using a bespoke battery of items assessing standard sociodemographic information and descriptive information particularly relevant to this sample (e.g. victim registry status, forced recruitment in the revolutionary armed forces). Socioeconomic status was defined according to monthly income relative to the national minimum wage in stratums from 1 ‘Below monthly minimum wage’ to 5 ‘More than eight times monthly minimum wage’.

*Potentially traumatic experiences* were screened using an adapted version of the Life Events Checklist (LEC-5; Weathers et al., 2013). The LEC-5 measures exposure to 17 events across one’s lifetime recognised as potentially traumatic in line with the DSM-5 A criterion for PTSD (APA, 2013). Examples include: ‘*Fire or explosion*’ and ‘*Life-threatening illness or injury*’. This measure was adapted to include five potentially traumatic experiences not included in the standardised measure, and deemed important to capture in the breadth of experiences of this particular group; ‘*Has a parent, romantic partner or family member repeatedly ridiculed you, put you down or told you that you were no good?*’, ‘*Have you experienced physical torture?*’, ‘*Have you experienced psychological torture?*’, ‘*Has your house or property ever been damaged?*’ and ‘*Have you ever experienced forcible displacement?*’. Responses to all items were recorded dichotomously (Yes/No).

*PTSD and CPTSD* symptomology was measured using the International Trauma Questionnaire (ITQ; Cloitre et al., 2018). The ITQ is a 18-item measure of symptomology in line with the ICD-11 classifications of PTSD and CPTSD (Cloitre et al., 2018; WHO, 2024b). Six items rate the experience of PTSD (*Re-experiencing*, *Avoidance*, and *Sense of threat*) in the previous 4 weeks, and six rate the experience of disturbances in self-organisation (*Affect Dysregulation*, *Negative Self Concept*, and *Interpersonal Disturbance*). These are each presented with three items assessing the presence of functional impairment in regard to social life, work, or daily activities. Diagnosis of probable PTSD is conferred through endorsement of one of each PTSD symptoms plus functional impairment. Probable diagnosis of CPTSD is conferred where the criteria for PTSD are met, plus endorsement of at least one of each disturbance in self-organisation plus functional impairment related to these, and supplants a probable diagnosis of PTSD (Cloitre et al., 2018).

*Identity orientation* was measured using the Social and Personal Identities Scale (SIPI; Nario-Redmond et al., 2004). The SIPI is a 16-item measure of the importance ascribed to one’s social (e.g. ‘*The similarity I share with others in my group(s)*’) and personal identity (e.g. ‘*My need to be completely distinct and unique from everyone else*’.). Each item is rated on a 7-point Likert scale from 1 ‘*Not at all important to who I am*’, to 7 ‘*Extremely important to who I am*’. Summary scores are imputed for the social and personal identity subscales by obtaining the mean for the eight items related to each, with greater scores indicative of greater importance or orientation ascribed to this domain of identity.

### Data Analysis

In accordance with the research questions proposed these data were analysed using Kruskal–Wallis/Chi-square tests reporting effect size (partial ETA-squared, Cramer’s V) to assess omnibus associations with probable diagnostic status according to ICD-11 criteria, and Wilcoxon/Proportion *Z*-Tests with Bonferroni corrections used for pairwise comparisons between probable diagnostic groups using the `*rstatix*` (Kassambara, 2023) and `*gtsummary*` (Sjoberg et al., 2021) packages in R (R Core Team, 2023). Primary analyses applied multinomial logistic regression using the `*nnet*` package (Ripley & Venables, 2022).

Multinomial logistic regression is a classification method that generalizes logistic regression to multiclass discrete outcomes, that is, in these analyses comparing three levels of diagnostic classification: No Diagnosis, PTSD, and CPTSD. In this case the degree of importance ascribed to social and personal identity orientation was regressed on to mutually exclusive probable diagnosis based on ICD-11 criteria (WHO, 2024b). Age, gender, socioeconomic status, and number of lifetime trauma experiences were included as covariates in the model as these are robustly evidenced to be associated with traumatic stress outcomes (Eide & Dyrstad, 2019; Kvedaraite et al., 2022; Ozer et al., 2003). Additionally, identification as a victim of armed conflict (i.e. victim registry status) was included as a potentially salient risk factor in this population (Lashkay et al., 2023; Tamayo-Agudelo & Bell, 2019). Copies of analysis syntax and results are made available in Supplementary File 1.

## Results

Applying the standardised scoring for the ITQ (see Cloitre et al., 2018) to the study sample: 22.74% (*n* = 123) received a probable diagnosis of PTSD, and 33.09% (*n* = 179) received a probable diagnosis of CPTSD. In preparation for primary analyses addressing the aims of this study, demographic and identity variable associations were assessed in relation to probable diagnostic status. Omnibus tests indicated no statistically significant difference to exist between groups on these characteristics (see Table 1).

**Table 1.** Sociodemographic information for study sample by probable diagnostic grouping.

Variable	Overall (*N* = 541)	No diagnosis (*N* = 239)	PTSD (*N* = 123)	CPTSD (*N* = 179)	*p*-value	Effect size
*Gender* ^2^					.602	0.050
Male	28.65% (155)	26.89% (64)	27.42% (34)	31.84% (57)		
Female	70.61% (382)	72.27% (172)	72.58% (90)	67.04% (120)		
Other gender identity	0.74% (4)	0.84% (2)	0.00% (0)	1.12% (2)		
Age^1^	44.11 (14.07)	43.02 (13.87)	44.35 (13.49)	45.37 (14.71)	.274	0.001
*Sexuality* ^2^					.477	0.084
Heterosexual	81.54% (433)	81.03% (188)	78.86% (97)	84.09% (148)		
Homosexual	1.69% (9)	2.16% (5)	0.81% (1)	1.70% (3)		
Bisexual	1.18% (10)	0.81% (1)	2.44% (3)	2.84% (3)		
I prefer not to answer	13.37% (71)	15.09% (35)	15.45% (19)	9.66% (17)		
Other sexuality	1.51% (8)	0.86% (2)	2.44% (3)	1.70% (3)		
*Ethnicity* ^2^					.389	0.102
Do not identify with any group	31.93% (129)	32.95% (57)	25.00% (24)	35.56% (48)		
Indigena (Indigenous)	19.80% (80)	16.18% (28)	25.00% (24)	20.74% (28)		
Hispano (Hispanic)	34.65% (140)	38.73% (67)	35.42% (34)	28.89% (39)		
I prefer not to answer	12.13% (49)	11.56% (20)	12.50% (12)	12.59% (17)		
Other ethnicity	1.49% (6)	0.57% (1)	2.08% (2)	2.22% (3)		
*Education* ^2^					.381	0.126
Baccalaureate	39.59% (213)	41.35% (98)	40.16% (49)	36.87% (66)		
Higher Education	3.90% (21)	3.36% (8)	6.56% (8)	2.79% (5)		
None	2.60% (14)	0.84% (2)	1.64% (2)	5.59% (10)		
Other qualification	0.56% (3)	0.42% (1)	0.00% (0)	1.12% (2)		
Preschool	1.67% (9)	1.68% (4)	1.64% (2)	1.68% (3)		
Primary	27.14% (146)	25.74% (61)	28.69% (35)	27.93% (50)		
Professional technical	5.39% (29)	6.72% (16)	4.10% (5)	4.47% (8)		
Technical	13.75% (74)	14.35% (34)	11.48% (14)	14.53% (26)		
Undergraduate	5.39% (29)	5.46% (13)	5.74% (7)	5.03% (9)		
*Economic status* ^2^					.096	0.094
Stratum 1–2 (very low – low)	98.13% (526)	99.15% (234)	95.90% (117)	98.31% (175)		
Stratum 3–4 (med. low – medium)	1.87% (10)	0.85% (2)	4.10% (5)	1.69% (3)		
*Registered as victim* ^2^					.907	0.031
Not registered, not a victim	3.90% (21)	4.20% (10)	2.44% (3)	4.49% (8)		
Not registered, but are a victim	9.28% (50)	9.66% (23)	8.94% (11)	8.99% (16)		
Registered	86.83% (468)	86.13% (205)	88.62% (109)	86.52% (154)		
Social identity^1^	4.39 (2.00)	4.46 (2.17)	4.23 (1.98)	4.41 (1.78)	.381	<0.001
Personal identity^1^	4.13 (1.94)	4.14 (2.16)	3.85 (1.82)	4.31 (1.70)	.119	0.004

*Note*. % (*n*); Mean (SD).

*Comparisons*: ^1^Kruskal–Wallis rank sum test; ^2^Pearson’s Chi-squared test.

In line with the first study aim the association between trauma events and probable diagnostic status were assessed (see Table 2). Results showed that those in the PTSD and CPTSD groups reported a greater mean number of cumulative lifetime traumas (η^2^ = .092, W = 51.376, *p* < .001). Those in the CPTSD group reported greater mean number of lifetime trauma exposure relative to both the No Diagnosis (ΔM = +2.9, *p* < .001) and PTSD groups (ΔM = +1.24, *p* < .001). Significant differences were found between diagnostic classifications in reporting of potentially traumatic experiences with the exception of exposure to *Toxic Substances*, *Physical Assault*, *Unwanted Sexual Contact*, and *Forced Displacement*. Effect sizes revealed that individual event endorsement had a negligible to small effect on probable diagnostic status with the most influential being exposure to *Severe Human Suffering* (η^2^ = .059), *Life Threatening Illness or Injury* (η^2^ = .036, and *Sudden Violent Death* (η^2^ = .034). Pairwise comparisons revealed only exposure to *Fire/Explosions*, *Psychological Torture*, and *Abuse/Ridicule From a Parent or Intimate Partner* were significantly associated with CPTSD diagnosis relative to PTSD diagnosis. Results of Chi-square comparison of index trauma endorsement, that is, experience was nominated as the worst, between groups was nonsignificant.

**Table 2.** Comparison of trauma event endorsements by probable diagnostic category.

Characteristic	Overall (*N* = 541)	No diagnosis (*N* = 239)	PTSD (*N* = 123)	CPTSD (*N* = 179)	Test statistic	*p*-val	Effect size	No diagnosis vs. PTSD	No diagnosis vs. CPTSD	CPTSD vs. PTSD
Total trauma endorsements^1^	8.27 (4.20)	6.92 (3.78)	8.60 (4.19)	9.84 (4.16)	51.947	<.001	0.093	<.001	<.001	.009
Natural disaster^2^	36% (197)	30% (71)	40% (50)	42% (76)	8.095	.017	0.011	.176	.031	>.999
Fire or explosion^2^	36% (194)	30% (72)	31% (38)	47% (84)	14.253	<.001	0.023	>.999	.002	.019
Traffic accident^2^	35% (190)	30% (72)	34% (42)	42% (76)	6.790	.034	0.009	>.999	.040	.496
Serious accident^2^	39% (212)	33% (79)	48% (59)	41% (74)	7.602	.022	0.010	.031	.325	>.999
Exposure to toxic substances^2^	17% (93)	14% (33)	17% (21)	22% (39)	4.512	.105	0.005	>.999	.141	>.999
Physical assault^2^	52% (284)	48% (114)	57% (71)	55% (99)	3.712	.156	0.003	.485	.343	>.999
Armed assault^2^	45% (243)	39% (93)	45% (56)	53% (94)	7.461	.024	0.010	.946	.025	.761
Sexual assault^2^	17% (93)	15% (35)	13% (16)	23% (42)	7.582	.023	0.010	>.999	.094	.095
Unwanted sexual contact^2^	13% (73)	12% (29)	15% (19)	14% (25)	0.739	.691	−0.002	>.999	>.999	>.999
Combat^2^	54% (291)	47% (112)	56% (70)	61% (109)	7.326	.016	0.012	.339	.021	>.999
Captivity^2^	42% (227)	35% (83)	46% (57)	49% (87)	8.969	.011	0.013	.156	.019	>.999
Illness or injury^2^	25% (134)	16% (38)	25% (32)	36% (64)	21.561	<.001	0.036	.105	<.001	.265
Severe human suffering^2^	54% (292)	40% (95)	64% (79)	66% (118)	33.951	<.001	0.059	<.001	<.001	>.999
Sudden violent death^2^	42% (227)	32% (76)	44% (55)	54% (96)	20.310	<.001	0.034	.080	<.001	.242
Sudden accidental death^2^	26% (138)	19% (45)	29% (36)	32% (57)	10.490	.006	0.015	.118	.010	>.999
Serious injury, harm, or death caused to another^2^	13.7% (20)	1.7% (4)	1.6% (2)	7.8% (14)	12.183	.002	0.020	>.999	.015	.103
Parent or partner ridicule^2^	36% (195)	29% (70)	31% (39)	48% (86)	16.859	<.001	0.028	>.999	<.001	.017
Physical torture^2^	18% (98)	12% (28)	23% (28)	23% (42)	11.590	.003	0.018	.033	.007	>.999
Psychological torture^2^	46% (247)	37% (87)	45% (56)	58% (104)	19.131	<.001	0.032	.420	<.001	.107
House or property damaged^2^	35% (187)	30% (71)	33% (41)	42% (75)	6.738	.027	0.009	>.999	.042	.453
Forced displacement^2^	89% (480)	89% (212)	86% (107)	90% (161)	1.030	.577	−0.002	>.999	>.999	>.999
Other stressful event^2^	37% (199)	27% (64)	44% (55)	45% (80)	17.889	<.001	0.029	.004	<.001	>.999
Index trauma^2^	—	—	—	—	41.649	.315	−0.003	—	—	—

*Note*. Continuous^1^: % (*n*), categorical^2^: mean (SD); *p*-val = alpha (*p*) value.

*Comparisons*: ^1^Kruskal–Wallis rank sum test; ^2^Pearson’s Chi-squared test; ^1,2^Pairwise proportion test comparisons using Bonferroni adjustment.

**Figure 1.**
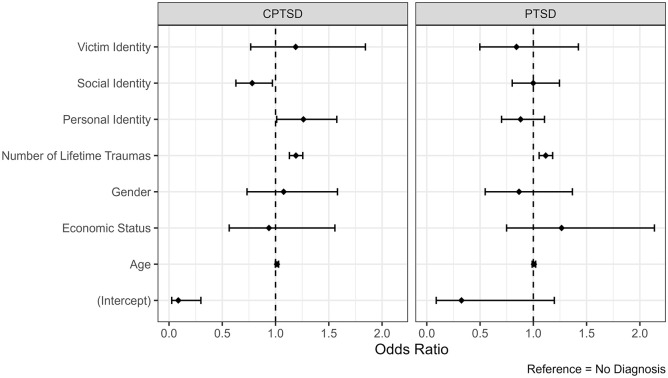
Results of multinomial logistic regression model of predictors for probable diagnostic status based on ITQ responses. *Note*: Odds ratios for each predictor are represented by point estimate, and the 95% confidence interval represented by the whiskers extending each point. The threshold of effect significance is represented by the vertical dashed line at the 1.0 x-intercept.

A multinomial logistic regression including identity variables and covariates previously evidenced to influence traumatic stress outcomes applied was found to provide adequate fit to these data: χ^2^(4) = 39.00, *p* < .001, McFadden pseudo-*R*^2^ = .14. Model results showed that cumulative lifetime trauma was associated with increased risk of screening positively for PTSD and CPTSD relative to the No Diagnosis group (see Table 3 and Figure 1). Within the model, social and personal identity orientation were found to be significant predictors of probable CPTSD diagnosis relative to No Diagnosis, however these associations were non-significant with PTSD diagnostic status relative to the No Diagnosis group. Self-reported social identity orientation was associated with reduced odds of probable CPTSD diagnostic status (OR = 0.79, 95% CI = 0.64–0.98, *p* = .035), and is thus suggested to be a resilience factor. In contrast, personal identity orientation was associated with increased odds of probable CPTSD diagnosis (OR = 1.25, 95% CI = 1.01–1.56, *p* = .044), and thus considered a risk factor for this outcome. Victim Identity was found not to significantly predict probable PTSD or CPTSD diagnostic status.

**Table 3.** Results of multinomial logistic regression model predicting probable diagnostic category.

Predictor	PTSD						CPTSD					
	Est	SE	Statistic	*p*-val	Low CI	High CI	Est	SE	Statistic	*p*-val	Low CI	High CI
(Intercept)	0.293	0.661	−1.854	.064	0.080	1.072	0.087	0.630	−3.881	<.001	0.025	0.298
Social identity	1.024	0.112	0.211	.833	0.823	1.274	**0.793**	**0.110**	**−2.108**	**.035**	**0.639**	**0.984**
Personal identity	0.883	0.114	−1.089	.276	0.706	1.105	**1.252**	**0.112**	**2.009**	**.044**	**1.006**	**1.560**
Victim identity	0.845	0.267	−0.630	.529	0.501	1.425	1.200	0.224	0.814	.416	0.773	1.863
Lifetime traumas	**1.119**	**0.029**	**3.889**	**<.001**	**1.057**	**1.184**	**1.193**	**0.027**	**6.560**	**<.001**	**1.132**	**1.258**
Economic status	1.291	0.265	0.966	.334	0.769	2.170	0.904	0.265	−0.381	.704	0.538	1.520
Gender	0.850	0.233	−0.699	.484	0.538	1.341	1.072	0.197	0.354	.724	0.729	1.578
Age	1.005	0.008	0.573	.567	0.988	1.022	1.013	0.008	1.667	.096	0.998	1.029

*Note*. Statistically significant results highlighted by bold text. Est = estimate; SE = standard error; *p*-val = alpha (*p*) value; CI = confidence interval.

## Discussion

This study offers a novel investigation of trauma exposure and identity orientation associated with PTSD and CPTSD in a trauma-exposed Latin American sample. The first study aim was to explore the association between trauma experiences and probable CPTSD diagnosis. Results showed that cumulative trauma experiences were associated with traumatic stress outcomes, and that greater cumulative trauma was associated with probable CPTSD diagnosis. This is in line with previous evidence suggesting that CPTSD is associated with exposure to greater numbers of potentially traumatic events across the life course, above traditional PTSD diagnosis (Cloitre et al., 2009; Hyland et al., 2017; Kira et al., 2022; Spikol et al., 2022).

Results further suggested that individual trauma experiences had relatively little effect distinguishing probable diagnostic outcomes. It was found that only exposure to *Fire/Explosions*, *Psychological Torture*, and *Abuse/ Ridicule From a Parent or Intimate Partner* were associated with elevated risk of CPTSD compared to PTSD. This partially supports theories of Complex Trauma that suggest that potentially traumatic experiences of an interpersonal nature are more likely to be associated with PTSD and CPTSD difficulties (Jowett et al., 2020). These results may likewise be related to the ‘Type-III’ trauma model, describing the risk for mental ill-health when potentially traumatic experiences are prolonged and often comprise multiple forms inclusive of interpersonal and non-interpersonal events (see Kira, 2010; Kira et al., 2022). This is particularly relevant in contexts effected by prolonged conflict where there is an increased risk of experiencing multiple and complex trauma events, and the risk for traumatic stress reactions is elevated (Greene et al., 2018). The results of the current study align with this suggesting that no individual trauma or type of trauma event predicts CPTSD in this post-accord context.

This study further aimed to assess orientation of social and personal identity, and identification with a victim group, as predictors of probable CPTSD diagnosis. Results indicated that greater social identity orientation was considered a resilience factor for CPTSD, and greater personal identity orientation was considered a risk factor. This aligns with the Memory and Identity Theory of CPTSD suggesting that social connectedness and compassion are protective against CPTSD outcomes (Hyland et al., 2023). In the current study it may therefore be considered that those placing greater importance on social group membership and interaction may draw on these resources more successfully and benefit from support in emotional regulation and reduced self-criticism that mitigate CPTSD development (see Hyland et al., 2023).

The finding of greater personal identity orientation to be a risk factor for CPTSD may be similarly understood through this theory; whereby greater propensity to be self-reliant places individuals at increased risk for mental ill-health as they forgo potential social support through group orientation, a robustly evidenced resilience factor for traumatic stress (Charuvastra & Cloitre, 2008; Hyland et al., 2023). Alternatively, it may be considered that the experience of trauma and CPTSD difficulties, particularly those disturbances in self-organisation characteristic of diagnosis, may be associated with disruptions to personal identity and sense of self which may be more distressing for those with a greater personal identity orientation (Møller et al., 2021). There is hence a need to further consider the effects of trauma on self-concept and self-efficacy in relation to CPTSD-related distress (Maercker et al., 2022).

It is suggested that experience of multiple trauma events or Complex Trauma has a ‘cascading’ effect on mental health, whereby these experiences contribute to maladaptive attachment and emotional regulation (Maercker et al., 2022). Included in this model is the hypothesis that trauma experiences and stress may contribute to social difficulties, ultimately driving association with CPTSD symptomology. The findings of the current study may therefore be considered through the lens of ‘social cure’ and ‘social curse’ (see Jetten et al., 2012) in understanding CPTSD difficulties. Interventions promoting greater individual orientation of social identity and cohesion may act a ‘social cure’ valuably mitigating CPTSD risk. As such, modular interventions where aspects of individual identity and regulation may be incorporated may be particularly suited to supporting those with CPTSD (see Karatzias & Cloitre, 2019; Karatzias et al., 2023).

Previous evidence has suggested that identification with a group potentially stigmatised or associated with trauma may be associated with increased risk of traumatic stress outcomes (Lashkay et al., 2023; Muldoon & Downes, 2007). Notably in the current study, identification with the Victim group was not associated with a significant risk for CPTSD difficulties. Previous research in other post-accord regions has demonstrated that while self-reported victim status is related to PTSD outcomes, even those who do not identify as a victim of armed conflict in the region are also likely to experience PTSD (Muldoon & Downes, 2007). Taken together it may be suggested that victimhood identity may not be sufficient to drive or categorise traumatic stress outcomes. In this respect the use of ‘victim’ labels in policy and services may adversely affect uptake by those in need of such provision as not all those who identify as victims need support and not all those who need support identify as victims. However as victim registry status or identity as a victim of the conflict was almost ubiquitous in this sample this assertion should be considered tentatively and warrants further investigation.

### Limitations

The current study offers a novel investigation of trauma and identity predictors of CPTSD in a trauma-exposed sample in Colombia; however these findings should be considered in light of some limitations.

Firstly, the current study analyses are cross-sectional in design and reliant on self-report assessment. While the measures used are standardised and validated (Fresno et al., 2023; Nario-Redmond et al., 2004) results should be interpreted with caution. Secondly, the study sample is drawn from a trauma-exposed community sample participating in a broader investigation of psychological trauma and mental health and thus results should not be considered indicative of the wider Colombian population. Future investigations may consider replication of these findings using representative and/ or clinical populations and assessment to further understand the role of trauma and identity in pathological development of CPTSD.

## Conclusions

This study furthers understanding of trauma and identity as predictors of CPTSD in a novel Latin-American context. Cumulative trauma is associated with reporting of CPTSD symptomology in this sample broadly in line with the Complex Trauma model of risk. Self-ascribed social identity orientation may be associated with resilience for this condition, as personal identity orientation is associated with increased risk of reporting CPTSD symptomology. Promoting social identity salience as a protective factor among those exposed to psychological trauma may be valuable in this context, however future research is required to better understand the interaction of trauma and identity with CPTSD.

## Supplemental Material

sj-html-1-isp-10.1177_00207640251318074 – Supplemental material for Trauma and identity predictors of ICD-11 PTSD and complex PTSD in a trauma-exposed Colombian sample Supplemental material, sj-html-1-isp-10.1177_00207640251318074 for Trauma and identity predictors of ICD-11 PTSD and complex PTSD in a trauma-exposed Colombian sample by Martin Robinson, Emanuele Fino, Gülseli Baysu, Rhiannon N Turner, Natasha I Bloch, Donncha Hanna and Chérie Armour in International Journal of Social Psychiatry
