# Supplementary material for: Trauma and identity predictors of ICD-11 PTSD and complex PTSD in a trauma-exposed Colombian sample
Source: Int J Soc Psychiatry. 2025 Feb 28;71(3):469–78. doi: 10.1177/00207640251318074 (PMC12012277; doi:10.1177/00207640251318074)
Supplement: sj-html-1-isp-10.1177_00207640251318074 – Supplemental material for Trauma and identity predictors of ICD-11 PTSD and complex PTSD in a trauma-exposed Colombian sample [file sj-html-1-isp-10.1177_00207640251318074.html]

Supplementary File 1


# Supplementary File 1

### R Markdown: Analyses and Results

#### 17 Mar 2024

### Data and Enironment Setup

```
rm(list=ls())
knitr::opts_chunk$set(echo = TRUE, collapse = TRUE, out.width = "100%",         # Standard Markdown chunk behaviour        
                      warning = FALSE, message = FALSE)                         # Streamline R Markdown output with removed messages

options(width = 1000)
```

```
library(haven)                                                                  # Package `haven` used for data read and display.
library(missMethods)                                                            # Package `missMethods` used for missing data analysis and imputation.
library(gtsummary)                                                              # Package `gtsummary` used for data summary and display.
library(tidyverse)                                                              # Package `tidyverse` used for data manipulation. 
library(rstatix)                                                                # Package `rstatix` used for uttility statistics.
library(flextable)                                                              # Package `flextable` used for data and results display.


setwd("D:/zb. Publication Records/gh. Social and Personal Identity Predict CPTSD")
data <- read.csv("D:/zb. Publication Records/gh. Social and Personal Identity Predict CPTSD/MIVIDA Methods Paper Data.csv")

data |> 
  dplyr::select(itq1:itqfi3, cptsd1:cptsdfi3, vic1, dem17, dem2, dem3, ntrauma, spis1:spis16) |> 
  naniar::mcar_test()                                                           # Data are MCAR on study variables
## # A tibble: 1 × 4
##   statistic    df p.value missing.patterns
##       <dbl> <dbl>   <dbl>            <int>
## 1     1683.  2019    1.00               59
  

missdata_est <- data |> 
  dplyr::select(record_id, itq1:itqfi3, cptsd1:cptsdfi3, vic1, dem17, dem2, dem3, ntrauma, spis1:spis16) |> 
  naniar::add_prop_miss()  |> naniar::add_any_miss()

missdata_est |> dplyr::select(any_miss_all) |> table()
## any_miss_all
## complete  missing 
##      437      125
  naniar::vis_miss(missdata_est)
```

```
# Custom functions for calculating effect sizes and pairwise tests.

Eff_size_r <- function(data, variable, by, ...) {                               # Calculate Wilcoxon R
                     rstatix::wilcox_effsize(data, as.formula(glue::glue("{variable} ~ {by}", ci = TRUE, conf.level = 0.95)))$effsize
}

Eff_size_V <- function(data, variable, by, ...) {                               # Calculate Cramer's V
                       table(data[[variable]], data[[by]]) |> 
                       rstatix::cramer_v()
}

Eff_size_eta <- function(data, variable, by, ...) {                             # Calculate eta^2
                     rstatix::kruskal_effsize(data, as.formula(glue::glue("{variable} ~ {by}", ci = TRUE, conf.level = 0.95)))$effsize
}
  
  
gts_pairwise.prop.test <- function(data, variable, by, ...) {                   # Calculate pairwise proportion comparisons
  tbl = table(data[[by]], data[[variable]])                                     # Use `gts_pairwise.prop.test` function to compare trauma reporting per diagnostic group.
  pairwise.prop.test(tbl, p.adjust.method = "bonferroni") |>  
    broom::tidy() |> 
    mutate(label = glue::glue("**{group2} vs. {group1}**")) |> 
    select(label, p.value) |> 
    spread(label, p.value)
}


apa_p.values <- function(x) {                                                   # Report p values in APA style
  style_pvalue(x, digits = 3) %>%
    stringr::str_replace("0.", ".")
}
```

```
### Missing Value Imputation using EM algorithm

d1 <-
missdata_est |> 
  dplyr::filter(prop_miss_all <= .20)

d2 <- d1 |> 
  dplyr::select(record_id, dem17, dem2, dem3, vic1, ntrauma, itq1:itqfi3, cptsd1:cptsdfi3, spis1:spis16) |> 
  missMethods::impute_EM(maxits = 1000)

d3 <- d2 |> 
  mutate_at(vars(itq1:cptsdfi3), 
  funs(case_when(. <= 0.4 ~ 0,
                 . <= 1.4 ~ 1, 
                 . <= 2.4 ~ 2,
                 . <= 3.4 ~ 3, 
                 . >= 3.5 ~ 4))) |> 
  mutate_at(vars(spis1:spis16), 
  funs(case_when(. <= 0.4 ~ 0,
                 . <= 1.4 ~ 1, 
                 . <= 2.4 ~ 2,
                 . <= 3.4 ~ 3, 
                 . <= 4.4 ~ 4,
                 . <= 5.4 ~ 5,
                 . <= 6.4 ~ 6,
                 . >= 6.5 ~ 7)))


studydata <- d3 |>                                                              # Recompile primary study variables with demographic data.
left_join(data, by = "record_id", suffix=c("",".y")) |> 
  select(-ends_with(".y"))
```

## Summary Statistics

```
studydata |>                                                                    # Relabel study variables for legibility.
rename(itq7 = itqfi1, itq8 = itqfi2, itq9 = itqfi3,                           
itqdso1 = cptsd1, itqdso2 = cptsd2, itqdso3 = cptsd3,
itqdso4 = cptsd4, itqdso5 = cptsd5, itqdso6 = cptsd6,
itqdso7 = cptsdfi1, itqdso8 = cptsdfi2, itqdso9 = cptsdfi3) |> 
mutate_at(vars("itq1", "itq2", "itq3", "itq4", 
"itq5", "itq6", "itq7", "itq8", 
"itq9", "itqdso1", "itqdso2", "itqdso3", 
"itqdso4", "itqdso5", "itqdso6", 
"itqdso7", "itqdso8", "itqdso9"), list(~ if_else(.x >= 2, true = 1, false = 0)))

df2 <- studydata |>                                                             # Calculate symptom endorsements and probable diagnosis.
  rowwise() |> 
mutate( Re      = sum(itq1, itq2, na.rm = T),
        Av      = sum(itq3, itq4, na.rm = T),
        Th        = sum(itq5, itq6, na.rm = T),
        PTSDfi  = sum(itqfi1, itqfi2, itqfi3, na.rm = T), 
        AD      = sum(cptsd1, cptsd2, na.rm = T),
        NSC     = sum(cptsd3, cptsd4, na.rm = T),
        DR      = sum(cptsd5, cptsd6, na.rm = T),
        DSOfi       = sum(cptsdfi1, cptsdfi2, cptsdfi3, na.rm = T)) |> 
mutate( Re_case         = ifelse(Re > 0, 1, 0),
        Av_case         = ifelse(Av > 0, 1, 0),
        Th_case         = ifelse(Th > 0, 1, 0),
        PTSDfi_case = ifelse(PTSDfi > 0, 1, 0),
        AD_case         = ifelse(AD > 0, 1, 0),
        NSC_case        = ifelse(NSC > 0, 1, 0),
        DR_case         = ifelse(DR > 0, 1, 0),
        DSOfi_case  = ifelse(DSOfi > 0, 1, 0),
        PTSD_diag       = ifelse(Re_case + Av_case + Th_case + PTSDfi_case == 4, 1, 0),
        CPTSD_diag  = ifelse(PTSD_diag + AD_case + NSC_case + DR_case + DSOfi_case == 5, 2, 0),
        ITQ_diag        = ifelse(CPTSD_diag == 2, 2, ifelse(PTSD_diag == 1, 1, 0)))

itq <- studydata |> dplyr::select('itq1':'cptsdfi3')                            # Calculate internal reliability (cronbach.alpha) for International Trauma Questionnaire.
ltm::cronbach.alpha(itq, na.rm = TRUE)
```

```
# Label probable diagnoses and calculate summary scores for Social and Personal Identity importance.
df3 <- df2 |> mutate(ITQ_diag.factor = recode_factor(ITQ_diag, "0" = "No Diagnosis", "1" = "PTSD", "2" = "CPTSD"),
                     SPIS_SI     = mean(c(spis1, spis3, spis5, spis7, spis9, spis11, spis13, spis15), na.rm = T),
                     SPIS_PI     = mean(c(spis2, spis4, spis6, spis8, spis10, spis12, spis14, spis16), na.rm =T))
  
##  Summary statistics for demographic variables
dem_table <- df3 |> 
  mutate(Gender          = dem2.factor,
         Sexuality       = dem6.factor,
         Age             = dem3,
         Ethnicity       = dem11.factor,
         Education       = dem14.factor,
         Economic_Stat   = dem17.factor,
         Victim_Registry = vic1.factor) |> 
      mutate(Gender = recode_factor(Gender, 
                                "Man" = "Male",
                                "Female" = "Female",
                                "Transgender man" = "Other Gender Identity",
                                "Transgender woman" = "Other Gender Identity",
                                "Transgender/gender  fluid" = "Other Gender Identity",
                                "Other" = "Other Gender Identity"),
         Ethnicity = recode_factor(Ethnicity, 
                            "Gitano" = "Other",
                            "Other - Please specify" = "Other"),
         Economic_Stat = recode_factor(Economic_Stat,
                                       "Stratum 1 (Very low)" = "Stratum 1-2 (Very Low - Low)",
                                       "Stratum 2 (Low)" = "Stratum 1-2 (Very Low - Low)",
                                       "Stratum 3 (Medium Low)" = "Stratum 3-4 (Medium Low - Medium)",
                                       "Stratum 4 (Medium)" = "Stratum 3-4 (Medium Low - Medium)"),
         Sexuality = recode_factor(Sexuality,
                                   "Heterosexual" = "Heterosexual")) |> 
  dplyr::select(ITQ_diag.factor, Gender, Sexuality, Age, Ethnicity, 
         Education, Economic_Stat, Victim_Registry, SPIS_SI, SPIS_PI) |> 
  tbl_summary(by = ITQ_diag.factor,
    label = list(Economic_Stat ~ "Economic Status",
                 Victim_Registry ~ "Registerd as Victim",
                 SPIS_SI ~ "Social Identity",
                 SPIS_PI ~ "Personal Identity"),
    type = c(Age, SPIS_SI, SPIS_PI) ~  "continuous" ,
    statistic = list(all_continuous() ~ "{mean} ({sd})",                        # Report all continuous variables: M (SD)
                     all_categorical() ~ "{p}% ({n})"),                         # Report all categorical variables: pct (n)
    digits = list(all_continuous() ~ 3,
                  all_categorical() ~ c(2,0)),
    missing = "no") |> 
    add_overall() |> 
    add_p(test = list(all_categorical() ~ "chisq.test",
                      all_continuous()  ~ "kruskal.test")) |>               
      modify_header(statistic ~ "**Test Statistic**")   |> 
  add_stat(fns = list(all_continuous()  ~ Eff_size_eta,                         # Calculate Effect sizes for comparisons
                      all_categorical() ~ Eff_size_V)) |> 
  modify_header(add_stat_1 ~ "**Effect Size**") |> 
  modify_fmt_fun(update = p.value ~ apa_p.values)

dem_table
```

| **Characteristic** | **Overall**, N = 5411 | **No Diagnosis**, N = 2381 | **CPTSD**, N = 1791 | **PTSD**, N = 1241 | **Test Statistic** | **p-value**2 | **Effect Size** |
| --- | --- | --- | --- | --- | --- | --- | --- |
| Gender |  |  |  |  | 2.739503 | .602 | 0.050 |
| Male | 28.65% (155) | 26.89% (64) | 31.84% (57) | 27.42% (34) |  |  |  |
| Female | 70.61% (382) | 72.27% (172) | 67.04% (120) | 72.58% (90) |  |  |  |
| Other Gender Identity | 0.74% (4) | 0.84% (2) | 1.12% (2) | 0.00% (0) |  |  |  |
| Sexuality |  |  |  |  | 7.569743 | .477 | 0.084 |
| Heterosexual | 81.54% (433) | 81.03% (188) | 84.09% (148) | 78.86% (97) |  |  |  |
| I prefer not to answer | 13.37% (71) | 15.09% (35) | 9.66% (17) | 15.45% (19) |  |  |  |
| Other - Please specify | 1.51% (8) | 0.86% (2) | 1.70% (3) | 2.44% (3) |  |  |  |
| Homosexual (gay or lesbian) | 1.69% (9) | 2.16% (5) | 1.70% (3) | 0.81% (1) |  |  |  |
| Bisexual | 1.88% (10) | 0.86% (2) | 2.84% (5) | 2.44% (3) |  |  |  |
| Age | 44.109 (14.067) | 43.021 (13.871) | 45.374 (14.714) | 44.371 (13.415) | 2.586334 | .274 | 0.001 |
| Ethnicity |  |  |  |  | 8.472896 | .389 | 0.102 |
| None - Does not identify with an ethnic group | 31.93% (129) | 32.95% (57) | 35.56% (48) | 25.00% (24) |  |  |  |
| Indigena | 19.80% (80) | 16.18% (28) | 20.74% (28) | 25.00% (24) |  |  |  |
| Hispanico | 34.65% (140) | 38.73% (67) | 28.89% (39) | 35.42% (34) |  |  |  |
| I prefer not to answer | 12.13% (49) | 11.56% (20) | 12.59% (17) | 12.50% (12) |  |  |  |
| Other | 1.49% (6) | 0.58% (1) | 2.22% (3) | 2.08% (2) |  |  |  |
| Education |  |  |  |  | 17.075444 | .381 | 0.126 |
| Baccalaureate | 39.59% (213) | 41.35% (98) | 36.87% (66) | 40.16% (49) |  |  |  |
| Higher Education | 3.90% (21) | 3.38% (8) | 2.79% (5) | 6.56% (8) |  |  |  |
| None | 2.60% (14) | 0.84% (2) | 5.59% (10) | 1.64% (2) |  |  |  |
| Other - Please specify | 0.56% (3) | 0.42% (1) | 1.12% (2) | 0.00% (0) |  |  |  |
| Preschool | 1.67% (9) | 1.69% (4) | 1.68% (3) | 1.64% (2) |  |  |  |
| Primary | 27.14% (146) | 25.74% (61) | 27.93% (50) | 28.69% (35) |  |  |  |
| Professional Technical Level | 5.39% (29) | 6.75% (16) | 4.47% (8) | 4.10% (5) |  |  |  |
| Technical | 13.75% (74) | 14.35% (34) | 14.53% (26) | 11.48% (14) |  |  |  |
| University undergraduate | 5.39% (29) | 5.49% (13) | 5.03% (9) | 5.74% (7) |  |  |  |
| Economic Status |  |  |  |  | 4.689688 | .096 | 0.094 |
| Stratum 1-2 (Very Low - Low) | 98.13% (526) | 99.15% (234) | 98.31% (175) | 95.90% (117) |  |  |  |
| Stratum 3-4 (Medium Low - Medium) | 1.87% (10) | 0.85% (2) | 1.69% (3) | 4.10% (5) |  |  |  |
| Registerd as Victim |  |  |  |  | 1.020504 | .907 | 0.031 |
| I am not registered because I am not a victim | 3.90% (21) | 4.20% (10) | 4.49% (8) | 2.44% (3) |  |  |  |
| I am not registered but I am a victim | 9.28% (50) | 9.66% (23) | 8.99% (16) | 8.94% (11) |  |  |  |
| Yes, I am registered | 86.83% (468) | 86.13% (205) | 86.52% (154) | 88.62% (109) |  |  |  |
| Social Identity | 4.388 (2.000) | 4.455 (2.169) | 4.407 (1.776) | 4.233 (1.976) | 1.928373 | .381 | 0.000 |
| Personal Identity | 4.131 (1.942) | 4.143 (2.155) | 4.311 (1.696) | 3.846 (1.824) | 4.257686 | .119 | 0.004 |
|  |  |  |  |  |  |  |  |
| --- | --- | --- | --- | --- | --- | --- | --- |
| 1 % (n); Mean (SD) | | | | | | | |
| 2 Pearson’s Chi-squared test; Kruskal-Wallis rank sum test | | | | | | | |

```
# gt::gtsave(as_gt(dem_table), "dem_table.rtf")
```

```
#  Summary statistics for Trauma Endorsement
trauma_endorsement_tab <- df3 |> 
  mutate(Gender          = dem2.factor,
         Sexuality       = dem6.factor,
         Age             = dem3,
         Ethnicity       = dem11.factor,
         Religion        = dem12.factor,
         Education       = dem14.factor,
         Economic_Stat   = dem17.factor,
         Living_Area     = dem8.factor,
         Victim_Registry = vic1.factor,
         FARC_Involved   = farc.factor,
         lec11a.factor   = recode_factor(lec11a.factor, "Yes, it happened to me" = "Yes", "Yes, it happened to a family member" = "Yes")) |> 
  dplyr::select(ITQ_diag.factor, ntrauma, lec1.factor, lec2a.factor, lec3a.factor, lec4a.factor,
         lec5a.factor, lec6a.factor, lec7a.factor, lec8a.factor,
         lec9a.factor, lec10a.factor, lec11a.factor, lec12a.factor,
         lec13a.factor, lec14a.factor, lec15a.factor, lec16a.factor,
         lec17a.factor, lec18a.factor, lec19a.factor, lec20a.factor,
         lec21a.factor, lec22a.factor, worsttrauma.factor) |> 
  tbl_summary(by = ITQ_diag.factor,
    label = list(lec1.factor = "Natural Disaster", lec2a.factor = "Fire or Explosion",
                 lec3a.factor = "Traffic Accident", lec4a.factor = "Serious Accident", 
                 lec5a.factor = "Exposure to Toxic Substances", lec6a.factor = "Physical Assault", 
                 lec7a.factor = "Armed Assault", lec8a.factor = "Sexual Assault",
                 lec9a.factor = "Unwanted Sexual Contact", lec10a.factor = "Combat", 
                 lec11a.factor = "Captivity", lec12a.factor = "Life Threatening Illness or Injury",
                 lec13a.factor = "Severe Human Suffering", lec14a.factor = "Sudden Violent Death", 
                 lec15a.factor = "Sudden Accidental Death", lec16a.factor = "Serious Injury, Harm, or Death Caused to Another",
                 lec17a.factor = "Parent or Partner Ridicule", lec18a.factor = "Physical Torture", 
                 lec19a.factor = "Psychological Torture", lec20a.factor = "House or Property Damaged",
                 lec21a.factor = "Forced Displacement", lec22a.factor = "Other Stressful Event",
                 worsttrauma.factor = "Index Trauma"),
    type = c(ntrauma) ~  "continuous", 
    statistic = list(all_continuous() ~ "{mean} ({sd})",
                     all_categorical() ~ "{p}% ({n})"),
    digits = all_continuous() ~ 2,
    missing = "no") |> 
    add_overall() |> 
    add_p(list(all_categorical() ~ "chisq.test",
               all_continuous() ~ "kruskal.test"),  include = everything()) |> 
  modify_header(statistic ~ "**Test Statistic**")   |> 
  add_stat(fns = list(everything()  ~ Eff_size_eta)) |> 
  add_stat(fns = list(all_categorical() ~ gts_pairwise.prop.test)) |> 
  modify_header(add_stat_1 ~ "**Effect Size**",
                add_stat_2 ~ "**Pairwise Comparisons**") |> 
  modify_fmt_fun(update = c(p.value, `**CPTSD vs. PTSD**`,`**No Diagnosis vs. CPTSD**`, `**No Diagnosis vs. PTSD**`) ~ apa_p.values)


df3 |> ungroup() |>                                                             # Pairwise comparison for `ntrauma` as continuous variable.
wilcox_test(ntrauma ~ ITQ_diag, alternative = "two.sided",
            p.adjust.method = "bonferroni")
## # A tibble: 3 × 9
##   .y.     group1 group2    n1    n2 statistic        p    p.adj p.adj.signif
## * <chr>   <chr>  <chr>  <int> <int>     <dbl>    <dbl>    <dbl> <chr>       
## 1 ntrauma 0      1        238   124    11200. 1.61e- 4 4.83e- 4 ***         
## 2 ntrauma 0      2        238   179    12692. 1.39e-12 4.17e-12 ****        
## 3 ntrauma 1      2        124   179     9176  1   e- 2 3.1 e- 2 *


trauma_endorsement_tab
```

| **Characteristic** | **Overall**, N = 5411 | **No Diagnosis**, N = 2381 | **CPTSD**, N = 1791 | **PTSD**, N = 1241 | **Test Statistic** | **p-value**2 | **Effect Size** | **CPTSD vs. PTSD** | **No Diagnosis vs. CPTSD** | **No Diagnosis vs. PTSD** | **Pairwise Comparisons** |
| --- | --- | --- | --- | --- | --- | --- | --- | --- | --- | --- | --- |
| ntrauma | 8.27 (4.20) | 6.92 (3.78) | 9.84 (4.16) | 8.60 (4.19) | 51.9467114 | <.001 | 0.093 |  |  |  |  |
| Natural Disaster | 36% (197) | 30% (71) | 42% (76) | 40% (50) | 8.0954764 | .017 | 0.011 | >.999 | .031 | .176 |  |
| Fire or Explosion | 36% (194) | 30% (72) | 47% (84) | 31% (38) | 14.2527563 | <.001 | 0.023 | .019 | .002 | >.999 |  |
| Traffic Accident | 35% (190) | 30% (72) | 42% (76) | 34% (42) | 6.7901400 | .034 | 0.009 | .496 | .040 | >.999 |  |
| Serious Accident | 39% (212) | 33% (79) | 41% (74) | 48% (59) | 7.6022153 | .022 | 0.010 | >.999 | .325 | .031 |  |
| Exposure to Toxic Substances | 17% (93) | 14% (33) | 22% (39) | 17% (21) | 4.5115161 | .105 | 0.005 | >.999 | .141 | >.999 |  |
| Physical Assault | 52% (284) | 48% (114) | 55% (99) | 57% (71) | 3.7115711 | .156 | 0.003 | >.999 | .485 | .343 |  |
| Armed Assault | 45% (243) | 39% (93) | 53% (94) | 45% (56) | 7.4607622 | .024 | 0.010 | .761 | .025 | .946 |  |
| Sexual Assault | 17% (93) | 15% (35) | 23% (42) | 13% (16) | 7.5815967 | .023 | 0.010 | .095 | .094 | >.999 |  |
| Unwanted Sexual Contact | 13% (73) | 12% (29) | 14% (25) | 15% (19) | 0.7388697 | .691 | -0.002 | >.999 | >.999 | >.999 |  |
| Combat | 54% (291) | 47% (112) | 61% (109) | 56% (70) | 8.3258626 | .016 | 0.012 | >.999 | .021 | .339 |  |
| Captivity | 42% (227) | 35% (83) | 49% (87) | 46% (57) | 8.9688129 | .011 | 0.013 | >.999 | .019 | .156 |  |
| Life Threatening Illness or Injury | 25% (134) | 16% (38) | 36% (64) | 26% (32) | 21.5605768 | <.001 | 0.036 | .265 | <.001 | .105 |  |
| Severe Human Suffering | 54% (292) | 40% (95) | 66% (118) | 64% (79) | 33.9508289 | <.001 | 0.059 | >.999 | <.001 | <.001 |  |
| Sudden Violent Death | 42% (227) | 32% (76) | 54% (96) | 44% (55) | 20.1302381 | <.001 | 0.034 | .424 | <.001 | .080 |  |
| Sudden Accidental Death | 26% (138) | 19% (45) | 32% (57) | 29% (36) | 10.0485118 | .007 | 0.015 | >.999 | .010 | .118 |  |
| Serious Injury, Harm, or Death Caused to Another | 3.7% (20) | 1.7% (4) | 7.8% (14) | 1.6% (2) | 12.7826176 | .002 | 0.020 | .103 | .015 | >.999 |  |
| Parent or Partner Ridicule | 36% (195) | 29% (70) | 48% (86) | 31% (39) | 16.8585193 | <.001 | 0.028 | .017 | <.001 | >.999 |  |
| Physical Torture | 18% (98) | 12% (28) | 23% (42) | 23% (28) | 11.5897763 | .003 | 0.018 | >.999 | .007 | .033 |  |
| Psychological Torture | 46% (247) | 37% (87) | 58% (104) | 45% (56) | 19.1309090 | <.001 | 0.032 | .107 | <.001 | .420 |  |
| House or Property Damaged | 35% (187) | 30% (71) | 42% (75) | 33% (41) | 6.7380427 | .034 | 0.009 | .453 | .042 | >.999 |  |
| Forced Displacement | 89% (480) | 89% (212) | 90% (161) | 86% (107) | 1.0299159 | .598 | -0.002 | >.999 | >.999 | >.999 |  |
| Other Stressful Event | 37% (199) | 27% (64) | 45% (80) | 44% (55) | 17.8890927 | <.001 | 0.029 | >.999 | <.001 | .004 |  |
| Index Trauma |  |  |  |  | 41.6490186 | .315 | -0.003 |  |  |  |  |
| A life-threatening illness or injury | 0.9% (4) | 1.0% (2) | 0% (0) | 1.9% (2) |  |  |  |  |  |  |  |
| A natural disaster (such as earthquakes, floods, volcanic eruptions, hurricanes, landslides) | 3.7% (17) | 4.4% (9) | 2.6% (4) | 3.9% (4) |  |  |  |  |  |  |  |
| Accident at work, at home or during recreational activities | 1.5% (7) | 1.5% (3) | 1.3% (2) | 1.9% (2) |  |  |  |  |  |  |  |
| Another stressful experience or event | 1.3% (6) | 0.5% (1) | 2.6% (4) | 1.0% (1) |  |  |  |  |  |  |  |
| Any other type of unwanted or unpleasant sexual activity | 1.1% (5) | 1.5% (3) | 1.3% (2) | 0% (0) |  |  |  |  |  |  |  |
| Armed assault (he has been shot, stabbed , threatened with a knife. Firearm or bomb) | 3.7% (17) | 1.5% (3) | 5.1% (8) | 5.8% (6) |  |  |  |  |  |  |  |
| Captivity (kidnapping , hostage-taking, prisoner of war, being deprived of liberty) | 2.2% (10) | 1.9% (4) | 2.6% (4) | 1.9% (2) |  |  |  |  |  |  |  |
| Combat or exposure to a war zone (such as military, combatant, or civilian) | 5.8% (27) | 5.3% (11) | 7.1% (11) | 4.9% (5) |  |  |  |  |  |  |  |
| Exposure to toxic substances | 0.4% (2) | 1.0% (2) | 0% (0) | 0% (0) |  |  |  |  |  |  |  |
| Forced displacement | 49% (230) | 55% (114) | 44% (69) | 46% (47) |  |  |  |  |  |  |  |
| House or property damaged | 0.2% (1) | 0.5% (1) | 0% (0) | 0% (0) |  |  |  |  |  |  |  |
| Physical assault (attack, robbery, beating, has been kicked or beaten) | 2.4% (11) | 2.4% (5) | 1.9% (3) | 2.9% (3) |  |  |  |  |  |  |  |
| Physical torture | 0.4% (2) | 0.5% (1) | 0.6% (1) | 0% (0) |  |  |  |  |  |  |  |
| Psychological torture | 2.6% (12) | 1.9% (4) | 4.5% (7) | 1.0% (1) |  |  |  |  |  |  |  |
| Severe suffering | 2.2% (10) | 1.5% (3) | 2.6% (4) | 2.9% (3) |  |  |  |  |  |  |  |
| Sexual assault (rape, attempted rape, forced to engage in any sexual act with use of force or threats ) | 4.3% (20) | 4.9% (10) | 4.5% (7) | 2.9% (3) |  |  |  |  |  |  |  |
| Traffic accident (includes accident of car, bus, boat or boat, train or plane ) | 3.0% (14) | 0.5% (1) | 6.4% (10) | 2.9% (3) |  |  |  |  |  |  |  |
| Unexpected accidental death | 1.5% (7) | 1.9% (4) | 1.3% (2) | 1.0% (1) |  |  |  |  |  |  |  |
| Violent death and unexpected (murder, suicide) | 12% (54) | 11% (22) | 10% (16) | 16% (16) |  |  |  |  |  |  |  |
| Your parents, partner or family member ridiculed repeatedly, verbally abused, or said you re not worth it | 1.9% (9) | 1.5% (3) | 1.3% (2) | 3.9% (4) |  |  |  |  |  |  |  |
|  |  |  |  |  |  |  |  |  |  |  |  |
| --- | --- | --- | --- | --- | --- | --- | --- | --- | --- | --- | --- |
| 1 Mean (SD); % (n) | | | | | | | | | | | |
| 2 Kruskal-Wallis rank sum test; Pearson’s Chi-squared test | | | | | | | | | | | |

```
# gt::gtsave(as_gt(trauma_endorsement_tab), "trauma_endorsement_tab.rtf")
```

```
#  Multinomial Logistic Regression, ITQ Diagnosis ~ Social Identity + Personal Identity
ITQDIAGregmodel   <- nnet::multinom(ITQ_diag.factor ~ SPIS_SI + SPIS_PI + vic1 +
                                    ntrauma + dem17 + dem2 + dem3, data = df3)  # Use package `nnet` to form multinomial model
## # weights:  27 (16 variable)
## initial  value 594.349248 
## iter  10 value 555.460442
## iter  20 value 541.993328
## iter  20 value 541.993325
## iter  20 value 541.993325
## final  value 541.993325 
## converged
OIM <- nnet::multinom(ITQ_diag.factor ~ 1, data = df3)                          # Use package `nnet` to form intercept only model for comparison
## # weights:  6 (2 variable)
## initial  value 594.349248 
## final  value 576.082436 
## converged

## Model fit indices
anova(OIM, ITQDIAGregmodel)                                                     # Likelihood ratio, sig.
## Likelihood ratio tests of Multinomial Models
## 
## Response: ITQ_diag.factor
##                                                      Model Resid. df Resid. Dev   Test    Df LR stat.      Pr(Chi)
## 1                                                        1      1080   1152.165                                   
## 2 SPIS_SI + SPIS_PI + vic1 + ntrauma + dem17 + dem2 + dem3      1066   1083.987 1 vs 2    14 68.17822 4.117988e-09
chisq.test(df3$ITQ_diag.factor, predict(ITQDIAGregmodel))                       # X^2 test
## 
##  Pearson's Chi-squared test
## 
## data:  df3$ITQ_diag.factor and predict(ITQDIAGregmodel)
## X-squared = 36.512, df = 4, p-value = 2.27e-07
pscl::pR2(ITQDIAGregmodel)                                                      # Nagelkerke == `r2cu`
## fitting null model for pseudo-r2
## # weights:  6 (2 variable)
## initial  value 594.349248 
## final  value 576.082436 
## converged
##           llh       llhNull            G2      McFadden          r2ML          r2CU 
## -541.99332458 -576.08243614   68.17822312    0.05917402    0.11840507    0.13437921


tbl1a <- broom::tidy(ITQDIAGregmodel,                                           # Format output using `broom` to `tidy` dataframe
                         conf.int = TRUE, conf.level = 0.95,                    # Request 95% CI
                         exponentiate = TRUE)                                   # Estimate == Odds Ratio,
flextable(tbl1a) |> colformat_double(digits = 3) #|> save_as_docx(path = "D:/zb. Publication Records/gh. Social and Personal Identity Predict CPTSD/multinomresults.docx")
```

| y.level | term | estimate | std.error | statistic | p.value | conf.low | conf.high |
| --- | --- | --- | --- | --- | --- | --- | --- |
| CPTSD | (Intercept) | 0.087 | 0.630 | -3.881 | 0.000 | 0.025 | 0.298 |
| CPTSD | SPIS\_SI | 0.793 | 0.110 | -2.108 | 0.035 | 0.639 | 0.984 |
| CPTSD | SPIS\_PI | 1.252 | 0.112 | 2.009 | 0.044 | 1.006 | 1.560 |
| CPTSD | vic1 | 1.200 | 0.224 | 0.814 | 0.416 | 0.773 | 1.863 |
| CPTSD | ntrauma | 1.193 | 0.027 | 6.560 | 0.000 | 1.132 | 1.258 |
| CPTSD | dem17 | 0.904 | 0.265 | -0.381 | 0.704 | 0.538 | 1.520 |
| CPTSD | dem2 | 1.072 | 0.197 | 0.354 | 0.724 | 0.729 | 1.578 |
| CPTSD | dem3 | 1.013 | 0.008 | 1.667 | 0.096 | 0.998 | 1.029 |
| PTSD | (Intercept) | 0.293 | 0.661 | -1.854 | 0.064 | 0.080 | 1.072 |
| PTSD | SPIS\_SI | 1.024 | 0.112 | 0.211 | 0.833 | 0.823 | 1.274 |
| PTSD | SPIS\_PI | 0.883 | 0.114 | -1.089 | 0.276 | 0.706 | 1.105 |
| PTSD | vic1 | 0.845 | 0.267 | -0.630 | 0.529 | 0.501 | 1.425 |
| PTSD | ntrauma | 1.119 | 0.029 | 3.889 | 0.000 | 1.057 | 1.184 |
| PTSD | dem17 | 1.291 | 0.265 | 0.966 | 0.334 | 0.769 | 2.170 |
| PTSD | dem2 | 0.850 | 0.233 | -0.699 | 0.484 | 0.538 | 1.341 |
| PTSD | dem3 | 1.005 | 0.008 | 0.573 | 0.567 | 0.988 | 1.022 |

```
#  Multinomial Logistic Regression Figure
fig2 <- tbl1a |> 
mutate(term = dplyr::recode(term, 
                      "vic1"    = "Victim Identity",
                      "SPIS_SI" = "Social Identity",
                      "SPIS_PI" = "Personal Identity",
                      "ntrauma" = "Number of Lifetime Traumas",
                      "dem3"    = "Age",
                      "dem2"    = "Gender",
                      "dem17"   = "Economic Status")) |> 
ggplot(aes(x=estimate, y=term, pch = term, label = term,
                  xmin = 0, xmax = 2)) + 
  geom_point(shape = 18, size = 2) + 
  geom_errorbar(aes(xmax = conf.high, xmin = conf.low, height = .01, width = 0.2)) +
  geom_vline(xintercept = 1, linetype = 2) +
  facet_grid(.~ y.level) +
  theme_bw() + theme(legend.position = "none") + labs(x = "Odds Ratio", y = NULL,
                                                      caption = "Reference = No Diagnosis")

fig2
```

```
# ggsave(plot = fig2, device = "png", width = 7, height = 4, dpi = 600, filename = "Fig2.png")
```

Assumption Checks for Multinomial Model
